# F-box protein 43 promoter methylation as a novel biomarker for hepatitis B virus-associated hepatocellular carcinoma

**DOI:** 10.3389/fmicb.2023.1267844

**Published:** 2023-11-02

**Authors:** Ying Zhang, Jing-Wei Wang, Xing Su, Jin-E Li, Xue-Fei Wei, Jie-Ru Yang, Shuai Gao, Yu-Chen Fan, Kai Wang

**Affiliations:** ^1^Department of Hepatology, Qilu Hospital of Shandong University, Jinan, China; ^2^Department of Hepatology, Qilu Hospital (Qingdao) of Shandong University, Qingdao, China; ^3^Hepatology Institute of Shandong University, Shandong University, Jinan, China

**Keywords:** hepatitis B virus-associated hepatocellular carcinoma, DNA methylation, MethyLight, FBXO43, diagnosis

## Abstract

**Background:**

Hepatocellular carcinoma (HCC) has a high prevalence and poor prognosis worldwide. Therefore, it is urgent to find effective and timely diagnostic markers. The objective of this study was to evaluate the diagnostic value of F-box protein 43 promoter methylation in peripheral blood mononuclear cells (PBMCs) for HCC.

**Method:**

A total of 247 participants were included in this study, comprising individuals with 123 hepatitis B virus-associated HCC, 79 chronic hepatitis B, and 45 healthy controls. F-box protein 43 methylation and mRNA levels in PBMCs were detected by MethyLight and quantitative real-time PCR.

**Result:**

F-box protein 43 promoter methylation levels were significantly lower in HCC PBMCs than the chronic hepatitis B (*P* < 0.001) and healthy control PBMCs (*P* < 0.001). Relative mRNA expression levels of F-box protein 43 in HCC PBMCs were significantly higher than those in chronic hepatitis B (*P* < 0.001) and healthy control PBMCs (*P* < 0.001). Receiver operating characteristic analysis of F-box protein 43 promoter methylation levels yielded an area under curve (AUC) of 0.793 with 76.42% sensitivity and 68.35% specificity when differentiating HCC from chronic hepatitis. These values for the F-box protein 43 promoter methylation level were superior to those of the alpha-fetoprotein serum (AFP) level (AUC: 0.780, sensitivity: 47.97%, and specificity: 96.20%), with increments in values for the combination of F-box protein 43 promoter methylation AFP levels (AUC: 0.888, sensitivity: 76.42%, and specificity: 86.08%).

**Conclusion:**

Hypomethylation of the F-box protein 43 promoter in PBMCs is a promising biochemical marker for HBV-associated HCC.

## Introduction

Primary liver cancer is a widespread cancer worldwide, with 906,000 new cases and 830,000 deaths, ranking sixth in incidence and third in mortality globally in 2020. Hepatocellular carcinoma (HCC) accounts for 75%−85% of primary liver cancer. In areas with high HCC prevalence, such as China and Korea, the main causes are chronic hepatitis B virus (HBV) infection, aflatoxin exposure, or both (Sung et al., 2021). According to the latest statistics, the 5-year survival rate of primary liver cancer is 21% (Siegel et al., 2023). Such poor outcomes are mainly because of the insidious onset of HCC, which is not easy to detect at an early stage, and most of them are discovered with a missed opportunity window for treatment. Alpha-fetoprotein (AFP) serum has been recognized as a non-invasive marker for the diagnosis of HCC. AFP serum level of 20 ng/ml is the upper limit for diagnosing HCC, but its sensitivity is 60%−70%. Moreover, AFP serum level is normal in 30%−40% of HCC patients (Trevisani et al., 2001; Gupta et al., 2003; Gopal et al., 2014). Additionally, some patients with chronic liver disease, especially those with a high degree of regeneration, may have elevated AFP even in the absence of malignant tumors (Di Bisceglie et al., 2005; Colli et al., 2006; Marrero et al., 2009; Lok et al., 2010). A report from the United States showed that AFP serum level was not elevated in 31% of patients diagnosed with HCC (Agopian et al., 2017). Therefore, a reliable and accurate non-invasive index is urgently needed for the early detection and diagnosis of HCC to alleviate the suffering of patients and improve the survival rate.

Methylation of deoxyribonucleic acid (DNA) cytosine-phosphate-guanine (CpG) islands is a very common epigenetic phenomenon in mammalian genomes for gene regulation. DNA methylation has been shown to affect biology in many ways, such as normal development, ribonucleic acid (RNA) and X-chromosome inactivation, imprinting, and development of tumors (Li et al., 1992, 1993; Panning and Jaenisch, 1998; Koch et al., 2018). DNA methylation of key regulatory regions has been shown to be a biomarker for tumor diagnosis and disease prognosis in many tumors, such as colorectal cancer, lung cancer, breast cancer, prostate cancer, and HCC (Salta et al., 2018; Constâncio et al., 2019; Nunes et al., 2019; Luo et al., 2020; Hernandez-Meza et al., 2021).

F-box protein 43 (FBXO43) is a member of the F-box protein family, consisting of approximately a 40-amino acid F-box motif. FBXO43 is involved in the biological processes of mitosis and meiosis (Schmidt et al., 2005; Gopinathan et al., 2017). In 2019, 10 genes, including FBXO43, were confirmed as prognostic and progression markers of HCC by gene coexpression network analysis (Xu et al., 2019). The prognostic value of FBXO43 in breast cancer has been evaluated histologically. The results showed that high expression of FBXO43 correlated positively with a high risk of metastasis and a poor prognosis (Vadhan et al., 2020). Moreover, studies have shown that the expression of FBXO43 is significantly increased in HCC cells and human tissues (Wu et al., 2023; Zhou et al., 2023). However, all of these reports were conducted at cellular and tissue levels. In clinical work, liver tissue is difficult to obtain, and the risk associated with surgery is high. Hematological tests are the most convenient and economical and do the least harm to the patient. However, as mentioned above, the detection rate of AFP serum level is not ideal. Therefore, we designed this experiment to investigate the value of FBXO43 promoter methylation as a non-invasive marker in the diagnosis of HCC using peripheral blood mononuclear cells (PBMCs) as a proxy for estimating the epigenetic rewiring potential of HBV infection.

In this study, we analyzed expression levels of FBXO43 to infer promoter methylation levels in PBMCs among patients with HBV-associated HCC, chronic hepatitis B (CHB), and healthy controls (HCs), as well as the clinicopathological features. At the same time, we evaluated the value of FBXO43 promoter methylation in PBMCs using MethyLight as a non-invasive marker in the diagnosis of HBV-associated HCC.

## Materials and methods

### Participants

**Figure 1:**
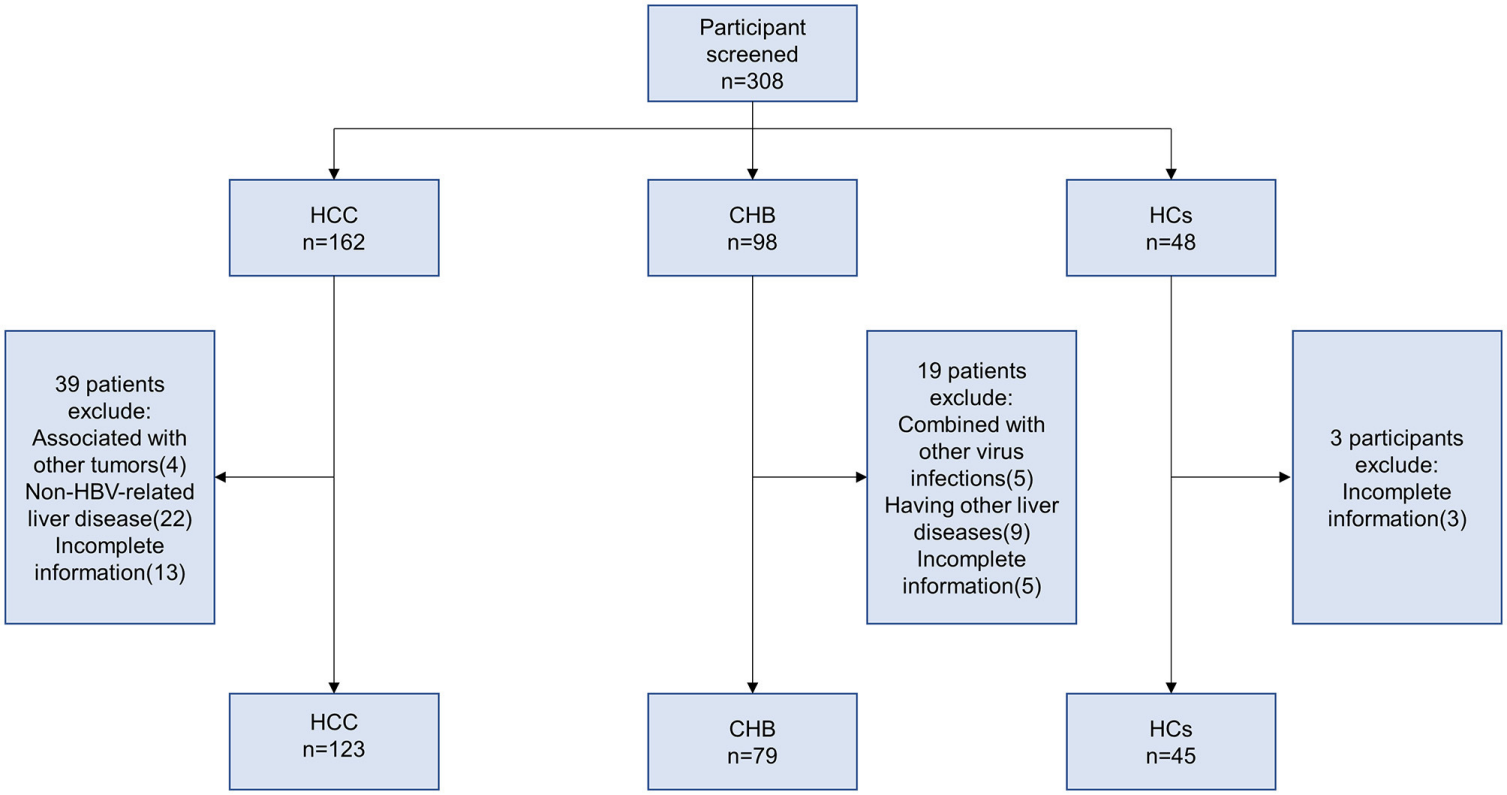
Flowchart for the enrollment of participants and the subset of samples used in the study.

In this study, 123 HBV-associated HCC patients, 79 CHB, and 45 HCs were recruited at the Department of Hepatology, Qilu Hospital of Shandong University, from January 2018 to December 2021. The diagnostic criteria for HBV-associated HCC were established according to the 2018 Practice Guidance by the American Association for the Study of Liver Diseases (AASLD) (Marrero et al., 2018). The inclusion criteria for CHB were in accordance with the AASLD 2018 Hepatitis B Guidance (Terrault et al., 2018). The exclusion criteria were as follows: (1) associated with other tumors; (2) combined with other virus infections (hepatitis A virus, hepatitis C virus, hepatitis D virus, hepatitis E virus, and human immune deficiency virus (HIV) infection); (3) other liver diseases (autoimmune hepatitis, alcoholic hepatitis, and drug hepatitis); and (4) incomplete information. The screening process is shown in Figure 1.

This study was approved by the local Research and Ethics Committee at Qilu Hospital of Shandong University in accordance with the 1975 Declaration of Helsinki. The study details were explained to the participants in detail, and their consent was obtained before enrollment.

### DNA extraction and sodium bisulfite modification

PBMCs were isolated by density gradient centrifugation with Ficoll-Paque (Pharmacia Diagnostics, Uppsala, Sweden) and stored at −80°C until use. Genomic DNA was extracted from PBMCs using a QIAamp DNA Blood Mini Kit (QIAGEN, Valencia, CA, USA) following the standard protocol for bisulfite conversion. DNA bisulfate modification was performed using an EZ DNA Methylation-Gold Kit (Zymo Research, Orange, CA, USA) according to the manufacturer's instructions. Finally, 20 μl of modified DNA was obtained for methylation.

### TaqMan probe-based quantitative methylation-specific polymerase chain reaction (MethyLight)

MethyLight was used to detect methylation levels of FBXO43 promoter and the promoter of the reference gene β-Actin. We used a website (http://genome.ucsc.edu/) to delineate the promoter of FBXO43 and another website (http://www.urogene.org/methprimer/) for sequence transformation. Then, oligo7 (OLIGO 1267 Vondelpark ColoradoSprings, CO 80907, USA) was used for the sequence design of probes and primers. Finally, the genome coordinates of FBXO43 are hg38, chr8:100133351–100145817. We selected the upstream 2,000 bp region of its TSS as the promoter region. Then, primers and probes were designed at 1,782–1,945 bp in the promoter region (Supplementary Figure S1). We found one CpG island, so only this one was tested. The sequence is shown in Table 1. The MethyLight reaction system consisted of a total of 10 μl volume, including 5 μl MethyLight Master Mix consisting of HotStarTaq Plus DNA Polymerase, EpiTect Probe PCR Buffer, and dNTP mix (dATP, dCTP, dGTP, dTTP), 0.4 μl forward primer, 0.4 μl reverse primers, 0.2 μl probe, 2 μl nuclease-free water, and 2 μl modified DNA. We used β-actin as the reference. The cycling conditions were 95°C for 15 min, followed by 45 cycles of 95°C for 15 s and 60°C for 60 s (Analytik Jena, Germany). SSSI methylase and bisulfite-modified human control DNA (QIAGEN, Hilden, Germany) were used as references for methylation. The MethyLight results PMR (percentage of methylated reference) were calculated using the following formula (Gao et al., 2015):


$$\begin{array}{c} PMR=\;100\%\times 2\;\exp\\ -[Delta\;Ct\;(target\;gene\;in\;sample-control\;gene\;in\;sample)\\ -Delta\;Ct\;(100\%\;methylated\;target\;in\;reference\;sample\\ -control\;gene\;in\;reference\;sample)]. \end{array}$$

**Table 1:** Primer and TaqMan probe sequences used to amplify bisulfite-converted DNA and RT-qPCR.

**Gene**	**Forward primer sequence (5^′^-3^′^)**	**Reverse primer sequence (5^′^-3^′^)**	**Probe oligo sequence**
**MethyLight**			
FBXO43	TTTTAAAGTGGGAATGGGGAGAAGTAGAGT	CCCGCAAACCTAAATCCTCGCTTAAAC	CCTCTCTCGCTCACCCCTACACCCGTCCCG
β-Actin	TGGTGATGGAGGAGGTTTAGTAAGT	AACCAATAAAACCTACTCCTCCCTTAAA	ACCACCACCCAACACACAATAACAAACACA
**RT-qPCR**			
FBXO43	GGAAAGTAAGCAGAAATTGGCGTG	GAGTGGCAGCATCCTCGACATT	
β-Actin	ATGGGTCAGAAGGATTCCTATGTG	CTTCATGAGGTAGTCAGTCAGGTC	

RT-qPCR, quantitative real-time PCR; FBXO43, F-box protein 43.

### RNA extraction and quantitative real-time PCR

RNA was extracted from PBMC cells using TRIzol (Invitrogen, Carlsbad, CA, USA).

We reverse-transcribed RNA to cDNA using a reverse transcription kit according to the instructions (ThermoFisher, Waltham, USA). Expression levels of FBXO43 and β-actin mRNA were detected using real-time PCR. This reaction system consisted of 10 μl, including 5 μl of TB Green premix (Takara, Shiga, Japan), 4.1 μl of nuclease-free water, 0.2 μl of forward primer, 0.2 μl of reverse primers, and 0.5 μl of cDNA. The cycling conditions were 95°C for 30 s, followed by 40 cycles of 95°C for 5 s, 55°C for 30 s, and 72°C for 60 s (Analytik Jena, Germany). The primer sequences used are shown in Table 1. The comparative method (2^−ΔΔCt^) was applied.

### Statistical analysis

Statistical analyses were performed with SPSS (version 26.0), MedCalc (version 20.010), and GraphPad Prism (version 8.0.1). Quantitative variables are expressed as the median (centile 25 and centile 75). Categorical variables are expressed as numbers (%). The Mann–Whitney *U*-test and the Kruskal–Wallis *H*-test were used to compare quantitative variables. A chi-square test was used to analyze categorical variables. Spearman's test was applied to determine the relationship between FBXO43 methylation level and quantitative clinical data. Receiver operating characteristic (ROC) curves were constructed to assess sensitivity, specificity, and respective areas under the curves (AUCs). Independent risk factors for HBV-associated HCC were analyzed by binary logistic regression. We considered *P* < 0.05 (two-sided) to indicate statistical significance.

## Results

### General characteristics

A total of 247 participants were enrolled in this study, including 123 HBV-associated HCC, 79 CHB, and 45 HCs. Their basic clinical characteristics are shown in Table 2. There were significant differences in PTA (*P* = 0.024), HBsAg (*P* < 0.001), and HBV-DNA (*P* < 0.001) between the HCC and CHB groups. Similarly, sex (*P* = 0.036), age (*P* < 0.001), ALT (*P* < 0.001), AST (*P* < 0.001), ALB (*P* < 0.001), TBIL (*P* < 0.001), PLT (*P* < 0.001), and AFP serum level (*P* < 0.001) were significantly different among the three groups.

**Table 2:** Baseline characteristics of participants.

**Variable**	**HCs (*n* = 45)**	**CHB (*n* = 79)**	**HCC (*n* = 123)**
Male, *n* (%)	30 (66.7)	53 (67.1)	100 (81.3)
Age (years)	49 (39–59)	42.00 (32.00–55.00)	55.00 (48.00–62.00)
ALT (U/L)	18 (13.50–24.50)	33.00 (21.00–102.00)	32.00 (19.00–59.00)
AST (U/L)	17 (15.00–22.00)	29.00 (20.00–74.00)	39.00 (24.00–74.00)
ALB (g/L)	47.00 (45.00–49.00)	46.80 (42.80–48.80)	41.30 (34.50–45.00)
TBIL (μmol/L)	12.50 (8.50–18.50)	13.80 (10.10–20.30)	17.60 (13.00–28.40)
PLT (10^9^/L)	236 (204.50–269.00)	182.00 (146.00–216.00)	154.00 (103.00–198.00)
PTA (%)	NA	93.00 (83.00–101.00)	87.00 (74.00–99.00)
AFP (ng/ml)	2.39 (1.76–3.06)	3.38 (2.18–11.99)	30.00 (5.71–800.00)
HBsAg (IU/ml)	NA	3,050.00 (967.12–12,630.80)	250.00 (212.70–2,026.13)
HBV-DNA (+), *n* (%)	NA	76 (96.2)	74 (60.2)

Quantitative variables are expressed as the median (25th percentile and 75th percentile). Categorical variables are expressed as the number (%). HCC, hepatocellular carcinoma; CHB, chronic hepatitis B; HCs, healthy controls; HBV, hepatitis B virus; AST, aspartate aminotransferase; ALT, alanine aminotransferase; ALB, albumin; TBIL, total bilirubin; PLT, blood platelet; PTA, prothrombin time activity; AFP, alpha-fetoprotein; HBsAg, hepatitis B s surface antigen; NA, not available.

### Hypomethylation of the FBXO43 promoter in patients with HBV-associated HCC

**Figure 2:**
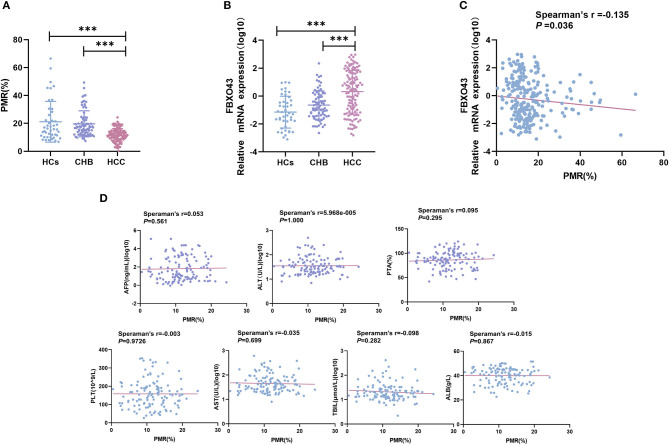
Relationships contrasting promoter methylation and expression levels of FBXO43 in PBMCs among participants, along with clinicopathological features. **(A)** FBXO43 methylation levels in PBMCs of HBV-associated HCC, CHB, and HCs. HCC: hepatocellular carcinoma; CHB: chronic hepatitis B; HCs: healthy controls (***P < 0.001). **(B)** FBXO43 mRNA levels in PBMCs of HBV-associated HCC, CHB, and HCs. HCC: hepatocellular carcinoma; CHB: chronic hepatitis B; HCs: healthy controls (***P < 0.001). **(C)** Relationships between FBXO43 promoter methylation levels and mRNA levels in PBMCs. **(D)** Relationships between the FBXO43 promoter methylation level and the quantitative clinical data in the HBV-associated HCC group.

MethyLight was used to detect the methylation status of FBXO43 promoter in PBMCs of HCC, CHB, and HC patients. The FBXO43 promoter methylation levels in HCC, CHB, and HCs are shown in Figure 2A. The methylation level of FBXO43 promoter in HCC was lower than that in CHB (*P* < 0.001, ANOVA) and HCs (*P* < 0.001, ANOVA), and the difference was statistically significant. There was no difference in the methylation level of the FBXO43 promoter between the CHB and HC groups (*P* = 0.641, ANOVA).

### FBXO43 mRNA levels in different groups

As methylation is a common mechanism that affects transcription, we examined the expression level of FBXO43 mRNA in PBMCs of the HCC, CHB, and HC groups, as shown in Figure 2B. The mRNA expression level of FBXO43 in the HCC group was significantly higher than that in the CHB (*P* < 0.001, ANOVA) and HC (*P* < 0.001, ANOVA) groups. There was no difference in the methylation level of the FBXO43 promoter between the CHB and HC groups (*P* = 0.103, ANOVA). The differences mentioned above for expression were statistically significant. To further clarify the relationship between the methylation level of FBXO43 promoter and the mRNA expression level, we used Spearman's rank correlation analysis to analyze the relationship. We found a weak negative but significant correlation between the FBXO43 promoter level and the mRNA expression level (Spearman's *r* = −0.135, *P* = 0.036; Figure 2C).

### Relationship between FBXO43 promoter methylation and clinicopathological features in HBV-associated HCC

The relationship between FBXO43 promoter methylation and clinicopathology was analyzed in HCC patients. As shown in Table 3, no significant differences were found upon comparing PMR to sex (*P* = 0.200), age (*P* = 0.281), HBV-DNA (*P* = 0.764), AFP serum level (*P* = 0.976), tumor number (*P* = 0.637), tumor size (*P* = 0.133), vascular invasion (*P* = 0.488), CTP staging (*P* = 0.992), or ascites (*P* = 0.317). Then, Spearman's rank correlation analysis was used to analyze the relationship between PMR and clinicopathological features, as shown in Figure 2D. FBXO43 promoter methylation also showed no correlation with AFP serum level (Spearman's *r* = 0.053, *P* = 0.561), ALT (Spearman's *r* = 5.968e−005, *P* = 1.000), PTA (Spearman's *r* = 0.095, *P* = 0.295), PLT (Spearman's *r* = −0.003, *P* = 0.9726), AST (Spearman's *r* = −0.035, *P* = 0.699), TBIL (Spearman's *r* = −0.098, *P* = 0.282), and ALB (Spearman's *r* = −0.015, *P* = 0.867).

**Table 3:** Associations between FBXO43 promoter methylation levels and clinicopathological features in HBV-associated HCC.

**Variable**	**Total number**	**PMR (%)**	***P*-value**
**Gender**			0.200^a^
Male	100	11.60 (9.03–14.73)	
Female	23	10.73 (7.76–12.46)	
**Age (year)**			0.281^a^
>50	84	10.89 (7.83–14.38)	
≤ 50	39	11.69 (9.55–14.76)	
**HBV-DNA**			0.764^a^
Negative	49	10.99 (7.56–14.60)	
Positive	74	11.44 (8.93–14.23)	
**AFP (ng/ml)**			0.976^a^
>20	67	11.66 (7.91–14.45)	
≤ 20	56	10.97 (9.08–14.46)	
**Tumor number**			0.637^a^
Single	65	10.96 (8.36–14.21)	
Multiple	58	11.54 (8.52–14.74)	
**Tumor size**			0.133^a^
≤ 5	79	10.88 (7.81–14.16)	
>5	44	12.01 (9.74–15.31)	
**Vascular invasion**			0.488^a^
Negative	81	10.96 (7.86–14.51)	
Positive	42	11.79 (9.13–14.29)	
**CTP staging**			0.992^b^
A	95	10.88 (8.10–14.06)	
B	19	13.21 (9.21–15.50)	
C	9	12.85 (10.15–18.18)	
**Ascites**			0.317^a^
No	88	11.60 (8.52–14.63)	
Yes	35	10.73 (7.91–13.30)	

a Mann–Whitney U-test.

b Kruskal–Wallis H-test.

CTP, Child–Turcotte–Pugh; PMR, percentage of methylated reference.

### Diagnostic value of the FBXO43 promoter methylation level

**Figure 3:**
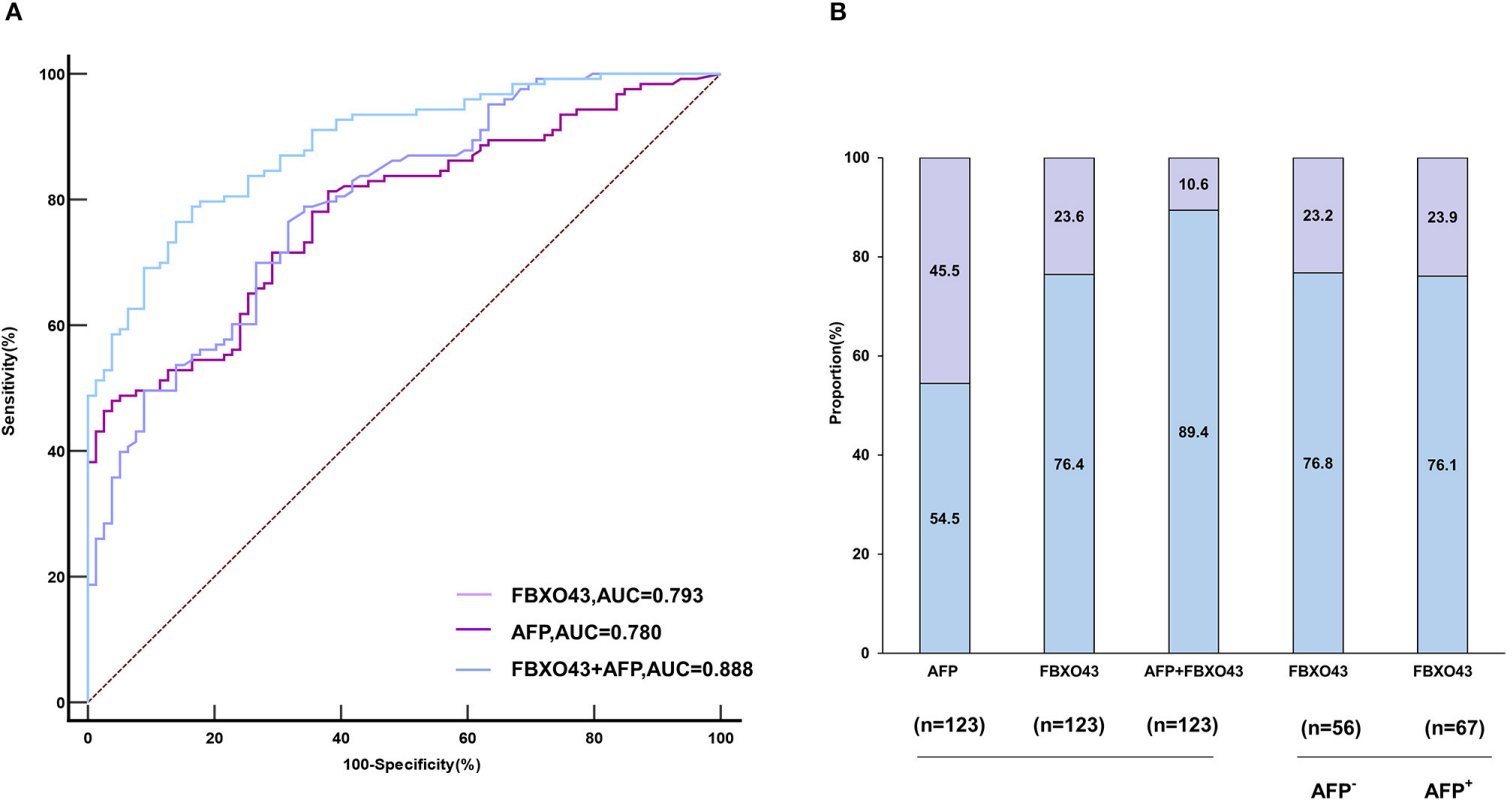
The diagnostic value of FBXO43 promoter methylation levels in PBMCs of HBV-associated HCC. **(A)** ROC curves of the PBMCs' FBXO43 promoter methylation level, AFP, and the combination of both in discriminating HBV-associated HCC from CHB. **(B)** Rate of positive results for AFP, PBMCs' FBXO43 promoter methylation levels, or both in patients with HBV-associated HCC, and for FBXO43 by AFP status.

ROC curves showed that the AUC of FBXO43 promoter methylation level (95% CI 0.730–0.846, AUC 0.793, sensitivity 76.42%, specificity 68.35%) was higher than that of AFP serum level (95% CI 0.717–0.835, AUC 0.780, sensitivity 47.97%, specificity 96.20%). In addition, the combination of FBXO43 methylation with AFP serum level improved the differentiation power (95% CI 0.836–0.928, AUC 0.888, sensitivity 76.42%, specificity 86.08%; Figure 3A, Table 4). A greater proportion of patients with HCC showed FBXO43 promoter methylation than increased AFP serum level [94 (76.4%) vs. 67 (54.5%) of 123 patients; Figure 3B]. Furthermore, 43 (76.8%) of 56 AFP serum level-negative patients with HCC had positive FBXO43 promoter methylation results. The rate was similar [51 (76.1%) of 67] in AFP serum level-positive patients. Moreover, when the FBXO43 promoter methylation level was combined with the AFP serum level, the rate of HCC diagnosis significantly increased to 110 of 123 (89.4%) in HCC (Figure 3B).

**Table 4:** Diagnostic value of FBXO43 promoter methylation and AFP in HBV-associated HCC.

	**Sensitivity (%)**	**Specificity (%)**	**Youden index**	**AUC**	**95% CI**
FBXO43	76.42	68.35	0.448	0.793	0.730–0.846
AFP	47.97	96.20	0.442	0.780	0.717–0.835
FBXO43 + AFP	76.42	86.08	0.625	0.888	0.836–0.928

AUC, area under curve; CI, confidence interval.

### Independent risk factors for HBV-associated HCC

Independent risk factors for HBV-associated HCC were assessed using univariate and multivariate analyses. The cohort was divided into two subgroups based on an optimal cutoff value of 14.56% FBXO43 promoter methylation level and 20 ng/ml AFP serum level. As illustrated in Table 5, the PMR value of FBXO43 promoter ≤ 14.56% [odds ratio (OR) = 9.373, 95% confidence interval (CI) 3.745–23.459, *P* < 0.001, multivariate logistic regression], male (OR = 3.125, 95% CI 1.159–8.424, *P* = 0.024, multivariate logistic regression), age (>50) (OR = 3.793, 95% CI 1.578–9.117, *P* = 0.003, multivariate logistic regression), HBsAg (>1,000 IU/ml, multivariate logistic regression; OR = 0.313, 95% CI 0.130–0.756, *P* = 0.010, multivariate logistic regression), HBV-DNA(+) (OR = 0.038, 95% CI 0.008–0.168, *P* < 0.001, multivariate logistic regression), and AFP serum level (>20 ng/ml; OR = 3.198, 95% CI 1.145–8.929, *P* = 0.027, multivariate logistic regression) were independent risk factors for HBV-associated HCC.

**Table 5:** Independent risk factors for the development of HBV-associated HCC.

**Variables**	**Univariate analysis**		**Multivariate analysis**	
	***P*** **-value**	**OR (95%CI)**	***P*** **-value**	**OR (95%CI)**
FBXO43 (PMR ≤ 14.56%)	< 0.001	7.001 (3.725–13.159)	< 0.001	9.373 (3.745–23.459)
Male	0.023	2.133 (1.111–4.096)	0.024	3.125 (1.159–8.424)
Age (>50)	< 0.001	3.742 (2.063–6.789)	0.003	3.793 (1.578–9.117)
HBsAg (>1,000 IU/ml)	< 0.001	0.189 (0.101–0.353)	0.010	0.313 (0.130–0.756)
HBV–DNA (+)	< 0.001	0.060 (0.018–0.200)	< 0.001	0.038 (0.008–0.168)
AFP (>20 ng/ml)	< 0.001	4.363 (2.293–8.303)	0.027	3.198 (1.145–8.929)
ALT (>50 U/L)	0.769	0.911 (0.491–1.693)	–	–
AST (>40 U/L)	0.116	1.589 (0.891–2.834)	–	–
PLT ( ≤ 100 × 10^9^/L)	0.001	7.816 (2.292–26.646)	0.062	4.455 (0.930–21.337)
TBIL (>7.1 μmol/L)	0.052	1.772 (0.996–3.151)	–	–
ALB ( ≤ 40 g/L)	< 0.001	3.824 (1.876–7.794)	0.514	1.463 (0.467–4.585)
PTA ( ≤ 60%)	0.068	0.145 (0.018–1.155)	–	–

OR, odds ratio.

## Discussion

Our study demonstrates that the methylation level of FBXO43 promoter in PBMCs of patients with HBV-associated HCC is significantly lower than that in CHB patients and HCs. Moreover, as a non-invasive marker for HBV-associated HCC, the methylation level of the FBXO43 promoter was more valuable than the AFP serum level. In particular, the positive rate of FBXO43 promoter methylation was also high in AFP serum level-negative patients, showing good complementarity with AFP serum level. Furthermore, the diagnostic value of the combination was higher. Finally, we found that FBXO43 promoter methylation, sex, age, HBsAg, HBV-DNA, and AFP serum level were independent risk factors for the occurrence and development of HBV-associated HCC. The correlation between FBXO43 promoter methylation and mRNA was Spearman's *r* = −0.135 with *P* = 0.036. A statistically significant association does not necessarily mean that the strength of the association is strong; however, the *P*-value suggests that there are fewer chances below 5% that this negative correlation with this intensity could have occurred by chance (Akoglu, 2018). Because the regulation mechanism of genes is very complex, here, we only observed a weak correlation between them, potentially due to the small number of subjects included in the study. As this study focused on the diagnostic value of FBXO43 promoter methylation for HCC, the specific relationship between FBXO43 methylation and mRNA was not specifically explored here, and we will explore the relationship between them in depth in the follow-up study.

DNA methylation plays an important role in gene expression regulation. Abnormal methylation is a marker of HCC development and is valuable in the early detection and prognosis of the disease (Nagaraju et al., 2022). Methylation is observed in precancerous lesions of varying degrees, such as cirrhosis, and changes in DNA methylation are also found during carcinogenesis (Kuramoto et al., 2017; Wijetunga et al., 2017). Methylation of many genes, such as ACADS (Chen et al., 2019), ADRA1A (Chen et al., 2020), BEX1 (Wang et al., 2021), and EYA4 (Hou et al., 2014), has been reported to be associated with HCC. Therefore, gene methylation assessment is a promising diagnostic tool for HCC. In addition, for the detection of methylation, most studies have adopted traditional methylation-specific PCR (MSP). This method avoids the use of restriction enzymes and their subsequent associated problems and is therefore highly sensitive. However, MSP is a qualitative method with relatively poor accuracy, strong subjectivity, and inconvenient analysis. In addition, if the distribution of 5-methylcytosine in the DNA to be tested is uneven, the detection will be more complicated, and false-positive results may occur. Other methods to detect the methylation of specific genes have also been applied in previous studies, such as pyrosequencing, bisulfite cloning, and sequencing. Although these two methods are highly accurate and can improve the sensitivity of detection, they require deep sequencing. Moreover, these two methods are technically complex, the operation process is cumbersome, and the price is expensive, which is not suitable for clinical application. Therefore, the methylation analysis technique we used is MethyLight, which is a fast, efficient, accurate, and qualitative experimental method for the analysis of molecular methylation levels. It can also analyze multiple samples rapidly at multiple gene loci. Moreover, there is no need for electrophoresis and hybridization after PCR, which reduces contamination and operation error. Its principle is based on PCR and TaqMan probe technology, which is highly sensitive and can detect minimal DNA in peripheral blood and other samples (Eads et al., 2000; Ogino et al., 2006). Hence, compared to the above methods, the advantages of MethyLight are evident.

It is necessary and urgent to search for simple and effective novel non-invasive diagnostic markers for HCC, a focus area of cancer research, because early detection of HCC can result in patients' valuable treatment opportunities. In recent years, there have been many studies on non-invasive markers, such as AFP serum level, AFP-L3, GP-73, DNA methylation, LncRNA, circRNA, and miRNA, among others (Peng et al., 2004; Zhang et al., 2016; Kisiel et al., 2019; Trevisani et al., 2019; Yu et al., 2020; Chalasani et al., 2021; Kim et al., 2021; Tayob et al., 2023). AFP is the most widely accepted non-invasive diagnostic marker. However, as mentioned above, the low sensitivity, high false-negative rate, and false-positive rate of AFP serum level limit its clinical application. Moreover, the use of AFP is no longer recommended in recent AASLD guidelines because of its low sensitivity (Terrault et al., 2018). Therefore, it is necessary to find novel non-invasive indicators to supplement or replace the AFP serum level. In China, ~70% of HCC is associated with HBV infection (de Martel et al., 2015). All patients included in this study were infected with HBV. In our study, the methylation of FBXO43 promoter in PBMCs showed high sensitivity in diagnosing HBV-associated HCC (76.42%), with a larger AUC than for AFP serum level (0.793 vs. 0.780), and better diagnostic performance and higher clinical application value when combined with AFP serum level. Moreover, PBMCs are easily available in clinical practice, requiring only a blood test with little harm to patients and a small cost.

At present, there are many methods for the diagnosis of HCC based on hematology, including circulating tumor DNA (ctDNA), circulating tumor cells (CTCs), exosomes, and PBMCs. However, ctDNA is easily degraded, has a short half-life, is not easy to preserve, has low specificity, and the amount of ctDNA in the peripheral circulation is extremely low, which is not easy to detect (Singh et al., 2020). CTCs also have the above problems as the content of CTCs in the peripheral circulation is very low, and CTCs have a short half-life, which make them difficult to detect. It is more suitable for guiding the prognosis of patients than for the early diagnosis of cancer (Danese et al., 2019; Deng et al., 2022). The application of exosomes in liquid biopsy is in the preliminary stage of exploration, and the diagnosis of cancer is found for early applications only in very few studies. Currently, the study of exosomal RNA is more common than exosomal DNA because RNA shows higher variations. In this study, we aimed to detect the methylation of FBXO43 promoter in PBMCs. PBMCs contain various cell types, such as natural killer cells (NK cells), monocytes, T and B lymphocytes, and dendritic cells (DCs), which have a positive response to tumor cells (Mosallaei et al., 2022). Compared with the above methods, PBMCs are easy to obtain, DNA is more stable, can be stored for a long time, and is more convenient for storage and retrospective analysis (Ziegler-Heitbrock, 2014). In addition, there was a clear difference between HCC and HC immune cell populations in PBMCs (Zhang et al., 2018; Han et al., 2021). Moreover, the stimulation of different external factors and pathological factors affects the regulation of target genes by target organs, and related genes in peripheral blood will also undergo the same changes (Mohr and Liew, 2007). Tumors may affect the epigenetic changes of immune cells in the circulatory system (Kristensen et al., 2012; Koch et al., 2018). DNA methylation in peripheral blood immune cells has recently been demonstrated in a variety of cancers, such as head and neck squamous cell carcinoma, colorectal cancer, and HCC (Huang et al., 2012; Zhang et al., 2018; Arayataweegool et al., 2019). Studies have shown that HBV infection can also cause DNA methylation changes. On the one hand, HBV can upregulate the expression of DNA methyltransferase genes (DNMTs), which leads to DNA methylation. On the other hand, HBV can regulate the methylation of immune genes and cause the methylation of related genes (Vivekanandan et al., 2010). Another study, a nested case-control study with 22 years of follow-up, showed that changes in HBV viral load caused changes in methylation at different sites (Kao et al., 2017). The patients included in this study had HBV-associated HCC, and the methylation of FBXO43 promoter in the HCC group was significantly lower than that in the CHB group. The methylation of FBXO43 promoter may also be affected by HBV infection. However, in this study, there was no significant difference in FBXO43 promoter methylation between the CHB and HC groups in the PBMCs. This may indirectly indicate that the FBXO43 promoter methylation status is not rewired by the HBV infection. Moreover, some studies have shown that the methylation profiles in immune cells are significantly different between the HBV infection stage and the HCC development stage (Zhang et al., 2018). In conclusion, HBV infection can cause the methylation of some genes in immune cells, but in some cases, the development of HCC may be due to the *de novo* methylation of another specific oncogene rather than the methylation that has already occurred at the time of HBV infection. Therefore, we speculated that FBXO43 promoter methylation was not directly associated with HBV infection. As this article is more focused on clinical research, mainly to find markers for early identification of HCC, future studies will be able to decode the epigenetic rewiring potential of HBV infection with an in-depth discussion of the mechanism determining the relationship between HBV infection and HCC in terms of FBXO43 promoter methylation. Overall, we found that FBXO43 promoter methylation can diagnose HBV-related HCC, but as a limitation of this study, we did not investigate its cause or development process and did not validate our conclusions regarding HCC caused by other etiologies, which we plan to explore in future studies.

Other limitations of this article are that this is a single-center and small-sample study. In the future, we will increase the sample size and conduct a multicenter study. Furthermore, we did not explore how FBXO43 promoter methylation affects the progression of HCC. We will continue to investigate the molecular mechanism of FBXO43 promoter methylation in the occurrence of HCC. Third, because it was difficult for us to obtain HCC tissues from the same group of patients, our conclusions were not verified in HCC tissues. In future studies, we will validate both HCC tissue and hematological assays to confirm our conclusion. Moreover, this study is only for the diagnosis of HCC by a single gene, and the combined detection of multiple genes may increase the detection rate of HCC and improve the sensitivity of biomarkers. In future studies, we will add the combined diagnosis of other differentially methylated genes to explore whether it can improve the diagnostic value of HCC. If other genes' promoters are methylated in PBMCs of HCC, then they can be used to diagnose HCC, where the combination with FBXO43 diagnosis may improve the diagnostic yield of HCC.

## Conclusion

The FBXO43 promoter methylation levels are significantly reduced in PBMCs of patients with HCC and can be investigated as a promising non-invasive biomarker for HCC diagnosis.

## Data availability statement

The raw data supporting the conclusions of this article will be made available by the authors, without undue reservation.

## Ethics statement

The studies involving humans were approved by Ethics Committee of Qilu Hospital of Shandong University. The studies were conducted in accordance with the local legislation and institutional requirements. The participants provided their written informed consent to participate in this study.

## Author contributions

YZ: Data curation, Formal analysis, Investigation, Methodology, Resources, Writing—original draft, Writing—review & editing. J-WW: Supervision, Writing—review & editing. XS: Investigation, Writing—review & editing. J-EL: Investigation, Writing—review & editing. X-FW: Formal analysis, Writing—review & editing. J-RY: Supervision, Writing—review & editing. SG: Supervision, Writing—review & editing. Y-CF: Supervision, Writing—review & editing. KW: Funding acquisition, Project administration, Supervision, Writing—review & editing.
