# Supplementary material for: F-box protein 43 promoter methylation as a novel biomarker for hepatitis B virus-associated hepatocellular carcinoma
Source: Front Microbiol. 2023 Nov 2;14:1267844. doi: 10.3389/fmicb.2023.1267844 (PMC10652413; doi:10.3389/fmicb.2023.1267844)
Supplement: Supplementary file 1 [file Table_1.docx]

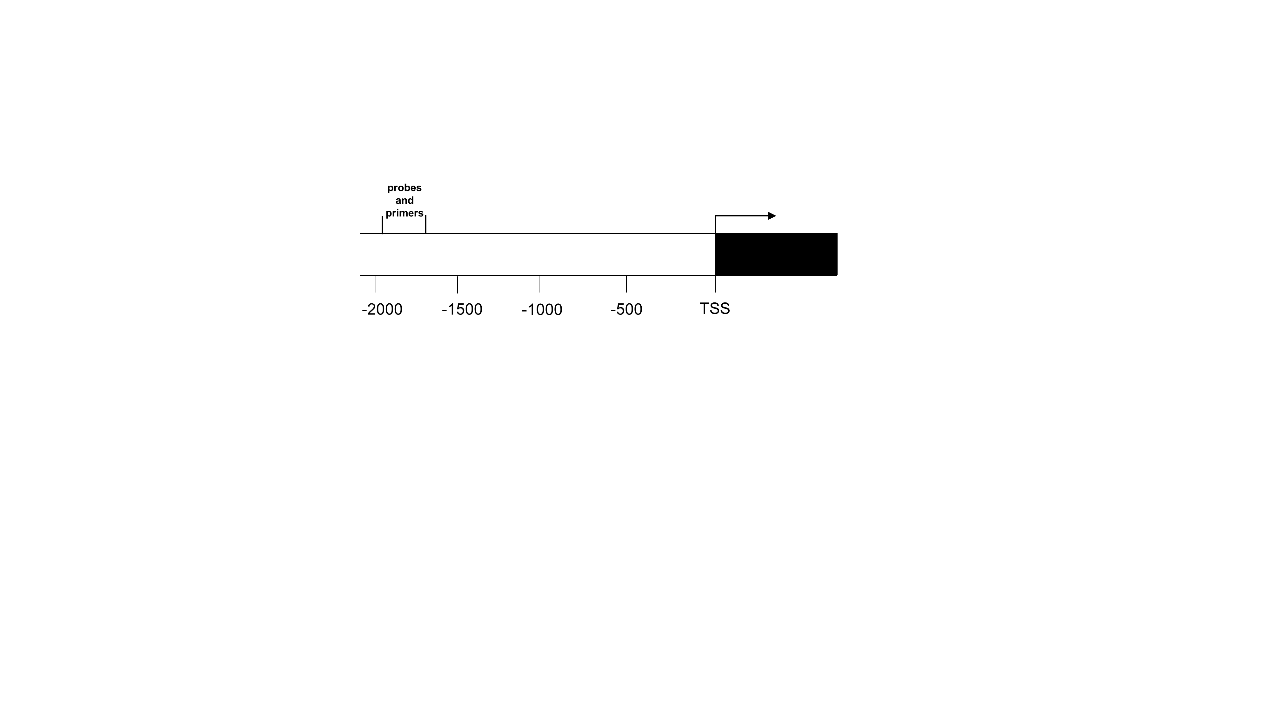


**Supplementary Figure 1** FBXO43 promoter methylation primer and probe design regions.

Keywords: TSS: transcription start site.
